# TEMPO-Nanocellulose/Ca^2+^ Hydrogels: Ibuprofen Drug Diffusion and In Vitro Cytocompatibility

**DOI:** 10.3390/ma13010183

**Published:** 2020-01-02

**Authors:** Andrea Fiorati, Nicola Contessi Negrini, Elena Baschenis, Lina Altomare, Silvia Faré, Alberto Giacometti Schieroni, Daniele Piovani, Raniero Mendichi, Monica Ferro, Franca Castiglione, Andrea Mele, Carlo Punta, Lucio Melone

**Affiliations:** 1 Department of Chemistry, Materials, and Chemical Engineering “G. Natta”—Politecnico di Milano, Piazza Leonardo da Vinci 32, I-20133 Milano, Italy; nicola.contessi@polimi.it (N.C.N.); baschenis.e@gmail.com (E.B.); lina.altomare@polimi.it (L.A.); silvia.fare@polimi.it (S.F.); monica.ferro@polimi.it (M.F.); franca.castiglione@polimi.it (F.C.); andrea.mele@polimi.it (A.M.); carlo.punta@polimi.it (C.P.); 2 INSTM, National Consortium of Materials Science and Technology, Local Unit Politecnico di Milano, 20133 Milano, Italy; 3 Istituto di Scienze e Tecnologie Chimiche (SCITEC-CNR), Via A. Corti 12, 20133 Milano, Italy; giacometti@ismac.cnr.it (A.G.S.); piovani@ismac.cnr.it (D.P.); rmendichi@libero.it (R.M.)

**Keywords:** TEMPO-oxidized nanocellulose, hydrogel, biomaterials, drug release, cytocompatibility

## Abstract

Stable hydrogels with tunable rheological properties were prepared by adding Ca^2+^ ions to aqueous dispersions of 2,2,6,6-tetramethylpiperidine 1-oxyl (TEMPO)-oxidized and ultra-sonicated cellulose nanofibers (TOUS-CNFs). The gelation occurred by interaction among polyvalent cations and the carboxylic units introduced on TOUS-CNFs during the oxidation process. Both dynamic viscosity values and pseudoplastic rheological behaviour increased by increasing the Ca^2+^ concentration, confirming the cross-linking action of the bivalent cation. The hydrogels were proved to be suitable controlled release systems by measuring the diffusion coefficient of a drug model (ibuprofen, IB) by high-resolution magic angle spinning (HR-MAS) nuclear magnetic resonance (NMR) spectroscopy. IB was used both as free molecule and as a 1:1 pre-formed complex with β-cyclodextrin (IB/β-CD), showing in this latter case a lower diffusion coefficient. Finally, the cytocompatibility of the TOUS-CNFs/Ca^2+^ hydrogels was demonstrated in vitro by indirect and direct tests conducted on a L929 murine fibroblast cell line, achieving a percentage number of viable cells after 7 days higher than 70%.

## 1. Introduction

Hydrogels gained tremendous interest in the biomedical field thanks to their high water content, tunable structural properties, cyto/biocompatibility and versatile properties. They are considered particularly appealing as drug-delivery systems and many applications can be found in various branches of medicine [1,2,3,4,5]. Moreover, the three-dimensional polymeric structure of hydrogels is also particularly suitable for the 3D culture of cells, as the mechanical, structural and compositional cues can guide cell fate and function. Three-dimensional cell culture in hydrogels has become an outstanding tool both for the fabrication of tissue engineered scaffolds [6] and for the development of 3D in vitro models [7]. The versatility of hydrogels allows for their fabrication, with embedded cells, by different technologies, including wet spinning [8], 3D printing [9], microspheres [10], and microfluidics [11]. In particular, injectable hydrogels have recently been gaining increasing interest regarding the localized introduction of small functional molecules for diagnostic purposes [12], for drug release and delivery [13] or injection of stem cells in regenerative therapy [14,15]. In this context, the investigation of the cyto/biocompatibility of novel hydrogels becomes crucial in order to propose them for biomedical applications.

Among possible natural-derived polymers used for the production of hydrogels for biomedical applications, cellulose nanofibers (CNFs) have emerged as an outstanding alternative, thanks to the high availability, low cost and good biocompatibility of cellulose [16,17,18,19,20]. A suitable method for the preparation of CNFs is based on the regioselective oxidation of the primary hydroxyls of cellulose to corresponding carboxylic groups, catalyzed by 2,2,6,6-tetramethylpiperidine 1-oxyl (TEMPO) in the presence of an NaBr/NaClO oxidizing system. Obtaining aqueous dispersions of nano-dimensioned fibers is facilitated by the use of a ultra-sonication process [20,21,22,23]. TEMPO oxidized and ultra-sonicated cellulose nanofibers (TOUS-CNFs) have been reported as versatile building blocks for the design of nanostructured materials with a wide range of potential applications [24,25,26,27,28,29,30,31,32].

Moreover, it has already been reported that aqueous dispersions of TOUS-CNFs exhibit interesting rheological properties, going from low viscous solutions to highly viscous gels as a function of fiber concentration [33,34]. The further addition of electrolytes (e.g., Al^3+^, Fe^3+^, Zn^2+^, Cu^2+^, Ca^2+^) into TOUS-CNFs dispersions screens the superficial charges leading to the reduction of mobility of the chain and, consequently, to the cross-linking between fibrils [34]. Under these conditions, gelation occurs rapidly and the mechanical and rheological properties of the gels obtained can be modulated by varying the cation nature and concentration [35,36]. Such hydrogels can find use in biomedicine for the controlled release of active principles [37], to enhance fibroblast adhesion [35,36], and for the preparation of macroporous hydrogel scaffolds supporting the growth of mouse fibroblast cells [38,39,40]. In this regard, TOUS-CNFs are particularly appealing in the biofabrication field. In fact, several works describe their use blended with other polymers (e.g., alginate [12,41], gelatin [42], pectin [43]) to enhance the printability of the (bio)inks and improve the shape fidelity of the printed scaffolds.

**Figure 1:**
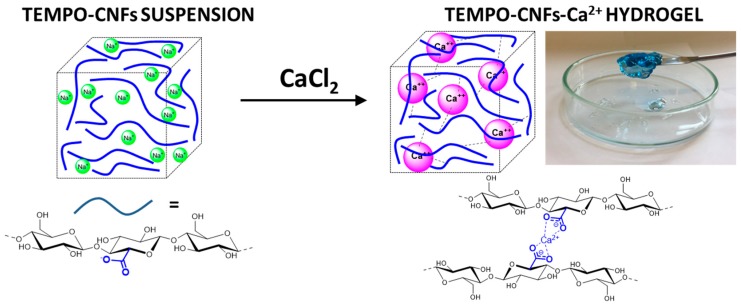
Production scheme of 2,2,6,6-tetramethylpiperidine 1-oxyl oxidized and ultra-sonicated cellulose nanofibers (TOUS-CNFs)-Ca^2+^ hydrogels.

Herein we report the preparation of stable and homogenous hydrogels from TOUS-CNFs aqueous suspensions cross-linked with calcium ions according to the scheme reported in Figure 1, and their use as cyto-compatible systems for controlled release of drugs that can be suitable as an injectable matrix. In a first step, we investigated the modification of the rheological properties of the hydrogels with the TOUS-CNFs content and Ca^2+^ concentration, in order to identify the formulation with the ideal elastic properties to be used for the delivery of drugs. Once the hydrogel was selected, we studied the diffusion into the system of a model drug (ibuprofen (IB), an anti-inflammatory drug often employed as model in drug release studies [4,29,44]), used both as pure molecule and as 1:1 complex with β-Cyclodextrins (β-CD) to slow down the drug path. These measures were conducted by diffusion-nuclear magnetic resonance (NMR) spectroscopy methods [45] combined with a high-resolution magic angle spinning (HR-MAS) setup to obtain high resolution data. Several applications of this novel technique to the investigation of soft materials [46,47], heterogeneous systems [48] and intact tissues [49] are reported in literature. The main strength of this approach is in providing detailed structural and dynamic information on the active molecules—IB in the present case—within the matrix actually used as scaffold. Finally, envisaging a possible application of the selected hydrogel, indirect cytotoxicity and direct in vitro cytocompatibility tests were conducted on a L929 murine fibroblast cell line.

## 2. Materials and Methods

Cellulose from fir pulp was kindly provided by Bartoli SPA (Carraia (Lu), Italy). Racemic ibuprofen as sodium salt and β-cyclodextrin (β-CD) were purchased from Sigma-Aldrich (St. Louis, MO, USA). All other chemicals were commercially available and were used without further purification. The morphological characterization of TOUS-CNFs was obtained by a transmission electron microscope (TEM, Philips CM 200, Koninklijke Philips N.V., Amsterdam, Netherlands) operating at 200 kV and equipped with a field emission gun filament.

### 2.1. Hydrogels Preparation and Characterization

#### 2.1.1. Synthesis

TEMPO-oxidized cellulose was prepared as described in literature [21,22]. Briefly, 100 g of cotton linters were suspended in water in a 1 L flask and homogenized with a domestic mixer. At mean time, TEMPO (2.15 g, 13.8 mmol) and KBr (15.42 g, 129 mmol) were dissolved in water (2 L) under magnetic stirring. The cellulose pulp was then added to the catalytic solution and more water was added in order to obtain a total volume of 5.7 L. NaClO solution (12.5% *w*/*w* aqueous solution—437 mL) was slowly added, while the pH value was maintained in the range of 10.5–11.0 by using a NaOH 4 M aqueous solution (250 mL). The reaction was left reacting overnight and then acidified to pH 1–2 with aqueous HCl (37% *w/w*). The white solid was filtered on a sintered glass funnel and washed extensively with deionized water (5 × 2 L). Final washes were performed with acetone (2 × 0.5 L) to remove water and allow rapid drying of the oxidized cellulose (84 g, 84% yield). The amount of carboxylic groups was determined by colorimetric titration with a NaOH solution using phenolphthalein as indicator. The measured oxidation degree was 1.51 mmol COOH/g cellulose.

TOUS-CNFs stock dispersions were prepared according to Table 1. The desired amount of TEMPO oxidized cellulose was weighed, 1 equivalent of NaOH 0.1 M with respect to the content of carboxylic groups was added, and the volume adjusted to 40 mL with water. Each mixture was sonicated at 0 °C for 20 min (Branson Sonifier 250 equipped with a 6.5 mm probe tip working at 20 kHz in continuous mode, with an output power 50% the nominal value (200 W)) until clear solutions were achieved. Finally, the pH of each sample was adjusted to 7.00 by the addition of small amounts of HCl_aq_ (0.01 M) or NaOH_aq_ (0.01 M).

TOUS-CNFs/Ca^2+^ hydrogel samples are listed in Table 2. They were prepared by adding the desired amount of water to 450 mg of the selected cellulose nanofibers stock dispersion and accurately mixing. Finally, the desired amount of an aqueous solution containing CaCl_2_ (100 mM) was added and the mixture and was gently mixed with a spatula, inducing the gelation process. After 30 min the hydrogel was ready for further analysis.

**C1** TOUS-CNFs/Ca^2+^ hydrogel loaded with IB was prepared as follows. IB (22.1 mg, 0.10 mmol) was dissolved in 300 μL of deionized water. This solution was added to 1350 mg of TOUS-CNFs aqueous dispersion (2% *w*/*v*, pH 7, stock solution C). The gelation was induced by the addition of 150 μL of Ca^2+^ 100 mM. The analogous hydrogels containing only β-CD (0.10 mmol) and only β-CD/IB (1:1) complex (0.10 mmol) were prepared in a similar way. All hydrogels were kept at rest at 25 °C for 12 h before HR-MAS measurements.

#### 2.1.2. Rheology

The rheological analysis was conducted by means of a stress-controlled AR2000 rotational rheometer (TA Instrument, New Castle, DE, USA) using cone-plate geometry: diameter D = 40 mm, angle α = 1°, polyacrylic material. To avoid increasing sample concentration during the experiment by solvent evaporation, a rheometer accessory named “solvent trap” was used. The temperature was maintained at 20 °C with a Peltier plate. Hydrogels rheological properties were determined using two different experiments: (i) oscillation curve or frequency (ω) sweep, ω from 0.1 rad/s to 628.3 rad/s with 5% of strain; (ii) flow curve or shear rate ($\dot{\gamma }$) sweep, $\dot{\gamma }$ from 0.1 s^−1^ to 1000 s^−1^. Strain value (5%) was chosen by optimizing the signal-to-noise ratio remaining in the linear range of the material.

#### 2.1.3. High-Resolution Magic Angle Spinning Nuclear Magnetic Resonance (HR-MAS NMR) Spectroscopy

The NMR spectra of the hydrogel systems were recorded on a Bruker Avance spectrometer operating at 500 MHz for the proton nucleus, equipped with a dual ^1^H/^13^C HR-MAS probe head for semisolid samples. Samples were transferred in a 4 mm ZrO_2_ rotor containing a volume of about 12 μL. ^1^H NMR experiments were performed at 305 K with the following acquisition parameters: time domain 16 K, relaxation delay 3 s, 8 scans and a spectral width of 8 ppm. Diffusion-ordered correlation spectroscopy (DOSY) experiments, based on a pulsed field gradient spin-echo (PGSE) approach, were performed using the bipolar pulse longitudinal eddy current delay (BPPLED) pulse sequence. In the z direction, the duration of the magnetic field pulse gradients (δ) and the diffusion times (Δ) were optimized for each sample to obtain complete dephasing of the signals with the maximum gradient strength. In each DOSY experiment, a series of 32 spectra with 32 K points were collected. For each experiment, 8 scans were acquired. For the investigated samples, Δ was set to 0.1 s, whereas the δ was set to 100 ms. The pulse gradients were increased from 2% to 95% of the maximum gradient strength in a linear ramp.

#### 2.1.4. In Vitro Characterization

In vitro tests were performed to investigate the stability and the suitability of **C1** hydrogel in sustaining viable cells encapsulation. For the in vitro tests, 4% *w*/*v* TOUS-CNFs hydrogel in water (stock solution D) was sterilized by autoclave [50] and used to prepare a 2% *w*/*v* hydrogel by mixing 1:1 in volume with culture medium, achieving **C1** hydrogel. The culture medium used for the in vitro tests was composed of Dulbecco’s modified Eagle medium (DMEM), 10% *v*/*v* bovine fetal serum (FBS), 1% *v*/*v* penicillin/streptomycin, 1 mM sodium pyruvate, 2 mM glutamine, and 10 mM HEPES.

In vitro stability tests were first performed to investigate the stability of the **C1** in culture medium at 37 °C (i.e., in vitro culture conditions); 500 µL of 2% *w*/*v* TOUS-CNFs hydrogel (n = 5 samples per time point) were gently placed in plastic Petri dishes (Ø = 35 mm) and 2 mL of CaCl_2_ 100 mM were used to crosslink TOUS-CNFs for 5 min, subsequently quickly washed twice with phosphate buffered saline (PBS). After washing, **C1** was immersed in 2 mL of complete culture medium and stored in incubator (37 °C, 5% CO_2_) for 7 days. At established time points (i.e., 30 min, 1, 2, 3, 4, 5 and 6 h, 1, 2, 3 and 7 days), the culture medium was removed and the **C1** hydrogel weight was recorded; at each time point, culture medium was replaced with fresh culture medium to simulate the cell culture conditions. Finally, the **C1** hydrogel percentage weight ratio was calculated following Equation (1) and reported in a function of time:

(1) $$Weight\;Ratio\;\left(\%\right)=\;\frac{W_{t}}{W_{0}}\times 100$$

where *W_t_* is the weight of **C1** at the time point *t* and *W_0_* is the weight of **C1** immediately after the crosslinking and washing with PBS, both hydrated.

In vitro biological tests were first performed by evaluating the indirect cytotoxicity of **C1**, according to ISO 10993-5 [51]. L929 murine fibroblasts cell line (ECACC No. 85011425) was selected as cell model. Culture medium eluates were prepared by placing 500 µL of **C1** in 24-multiwell tissue culture plastic (TCPS) and by immersing them in 1 mL of complete culture medium. Samples were then incubated (37 °C, 5% CO_2_) with culture medium for 1, 3 and 7 days to obtain the eluates; culture medium without samples was also incubated for the same time points as control. L929 cells were seeded in 96-multiwell (1 × 10^4^ cells per well) and cultured in 150 µL of complete culture medium until 70% confluence was reached. Then, culture medium was replaced with 150 µL of culture medium eluates or culture medium controls (n = 4 per time point) and cells well cultured in contact with eluates, or controls, for 24 h. After 24 h, the culture media were removed, replaced with 150 µL of a 10% *v*/*v* Alamar Blue^TM^ solution in culture medium and cells were incubated for 4 h (37 °C, 5% CO_2_). After 4 h, 100 µL were transferred from each well to a new 96-multiwell and fluorescence read by using a spectrophotometer (Tecan Genius Plus, λ_emission_ = 595 nm and λ_excitation_ = 540 nm). Finally, the percentage cell viability was calculated at each time point following Equation (2):

(2) $$Cell\;Viability\;\left[\%\right]=\frac{f_{sample}-f_{alamar\;blue}}{f_{control}-f_{alamar\;blue}}\times 100$$

where *f_sample_* is the fluorescence intensity measured for cells incubated with culture medium eluates, *f_control_* is the fluorescence measured for cells cultured with culture medium controls, and *f_alamar blue_* is the fluorescence of the Alamar Blue solution incubated without cells (i.e., background Alamar Blue solution fluorescence).

The possibility of embedding cells in **C1** was finally investigated; as comparison, alginate hydrogels were selected due to the similar divalent ion-driven crosslinking reaction to the one of TOUS-CNFs. In particular, a solution of 4% *w*/*v* alginate (medium viscosity, Sigma Aldrich) in water was prepared and mixed 1:1 in volume with culture medium to obtain a final 2% *w*/*v* alginate solution. A L929 cell suspension (1 × 10^6^ cells mL^−1^) was gently mixed to the TOUS-CNFs or alginate solution, subsequently crosslinked for 5 min in 100 mM CaCl_2_ solution; TCPS was seeded (2 × 10^4^ cell/well) and used as bi-dimensional control. Cells were cultured (37 °C, 5% CO_2_) for 7 days, by changing the culture medium every two days. A LIVE/DEAD staining was performed after 1, 3 and 7 days to check cell viability. At each time point, samples were washed with PBS, incubated for 40 min with the LIVE/DEAD staining solution (1 µM Calcein-AM, 10 µM propidium iodide in culture medium without FBS), washed twice with culture medium and observed by fluorescence microscopy (Zeiss Axioplan) to check the presence of viable (green) and dead (red) cells. Cells were then counted (n = 6 images per time point, per sample; ImageJ software) and the percentage of viable cell was calculated following Equation (3) [9]:

(3) $$Viable\;Cell\;\left[\%\right]=\frac{N_{viable\;cells}}{N_{viable\;cells}+N_{dead\;cells}}\times 100$$

## 3. Results and Discussion

### 3.1. Rheological Properties

**Figure 2:**
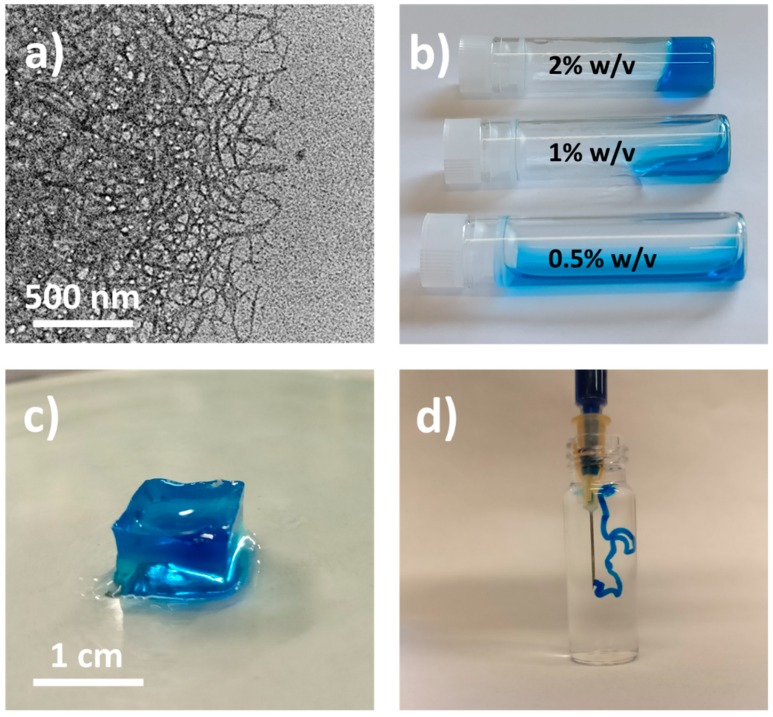
(**a**) Transmission electron microscope (TEM) image of TOUS-CNFs (scale bar: 500 nm); (**b**) Qualitative rheological behaviour of TOUS-CNFs aqueous suspensions at different CNF concentrations (i.e., 0.5, 1 and 2% *w*/*v*); (**c**) example of a cubic-shape TOUS-CNFs/Ca^2+^ hydrogel **C1** (scale bar: 1 cm); (**d**) extrusion of TOUS-CNFs aqueous dispersion (2% *w*/*v*) into Ca^2+^ (100 mM) aqueous solution (indigo dye used to improve the visualization).

The TOUS-CNFs obtained are of about 10 nanometers width and a length in the range of 200–400 nm (Figure 2a), in line with data reported in the literature [15,16]. It has been reported how aqueous dispersions of these nanofibers exhibit rheological properties typical of thixotropic materials [28]. By varying TOUS-CNFs concentrations, the rheological behavior moves from low viscous suspensions to highly viscous gels when increasing the amounts of dispersed nanofibers (Figure 2b). In spite of this gel-like character, the structure can be easily disrupted by high shear rate, leading to a viscous liquid capable to change, reversibly, from elastic-dominated to viscous-dominated regimes [33]. Thanks to the presence of the carboxylate groups on the fibers surface, TOUS-CNFs are able to interact with electrolytes dissolved in water. In particular, polyvalent electrolytes (e.g., Ca^2+^) can act as cross-linkers between fibers, reducing their mobility and consequently inducing a non-reversible gelation (Figure 2c). This gelation occurs rapidly (Figure 2d) and the mechanical and rheological properties of the hydrogel formed can be tuned by varying the cation nature and concentration [35,36].

**Figure 3:**
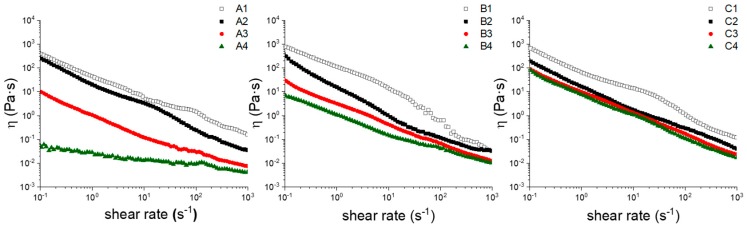
Dynamic viscosity (η, Pa·s) as a log-log function of shear rates at different TOUS-CNFs and Ca^2+^ concentrations. A: TOUS-CNFs 0.5% *w*/*v*; B: TOUS-CNFs 1.0% *w*/*v*; C: TOUS-CNFs 2% *w*/*v*. Symbols: □ [Ca^2+^] = 10 mM; ■ [Ca^2+^] = 5 mM; ● [Ca^2+^] = 2 mM; ▲ [Ca^2+^] = 0 mM.

Figure 3 shows the dynamic viscosity curve, η* = ƒ($\dot{\gamma }$), from flow rheological tests of four samples with CaCl_2_ concentration ranging from 10 mM (1) to 0 mM (4) and TOUS-CNFs concentration (*w*/*v*) of 0.5% (A), 1.0% (B), 2.0% (C). It appears clearly that dynamic viscosity values increase considerably as the CaCl_2_ concentration increases. Similarly, pseudoplastic rheological behaviour increases when either CaCl_2_ or TOUS-CNFs concentrations are increased. This behaviour demonstrates how CaCl_2_ is able to promote cross-linking and formation of stable hydrogels (“strong hydrogels”).

**Figure 4:**
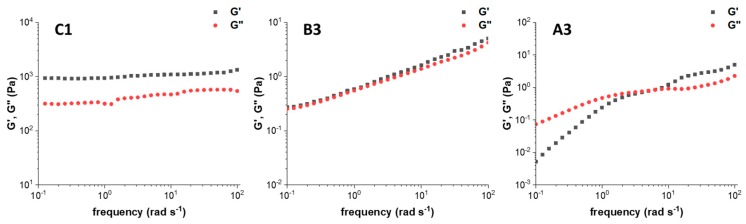
G’ (elastic) and G’’ (viscous) modules as a function of frequency of samples: C1 (TOUS-CNFs = 2.0%, CaCl_2_ = 10 mM); B3 (TOUS-CNFs = 1.0%, CaCl_2_ = 2 mM); A3 (TOUS-CNFs = 0.5%, CaCl_2_ = 2 mM).

Figure 4 shows a comparison of storage (G’) and loss (G”) modules as a function of the frequency from oscillation rheological tests of three samples: **C1** (TOUS-CNFs = 2.0%, CaCl_2_ = 10 mM); **B3** (TOUS-CNFs = 1.0%, CaCl_2_ = 2 mM); **A3** (TOUS-CNFs = 0.5%, CaCl_2_ = 2 mM). The elastic modulus G’ describes the solid-like behaviour of TOUS-CNFs gels, whereas the viscous modulus G” defines the liquid-like behaviour of the material. From sample **C1** (left) to sample **A3** (right) the rheological plots show behaviour of “**C1** strong-hydrogel”, “**B3** weak-hydrogel” and “**A3** macromolecules solution”. Differences between samples (gel structure) mainly depends on the cross-linking level and, to a lesser extent, from TOUS-CNFs concentration.

Oscillation rheological curves confirm that hydrogels formation (from solution to weak and strong) mainly depends on the CaCl_2_ concentration and also to a lesser extent on the TOUS-CNFs concentration. In particular, in sample C1, G’ and G” values are non-intersecting, with G’ always dominant over G”. This indicates that the elastic behavior dominates over the viscous one and the hydrogel is acting consistently solid-like, with strong stability. For this reason, **C1** resulted to be the ideal gel for our purpose and was selected for further investigation.

### 3.2. Ibuprofen Drug Diffusion in Hydrogels

Studies on the delivery of drugs from hydrogels are generally carried out by the determination of release kinetics in suitable aqueous media. However, several authors pointed out the limits of the reliability of such experiments because the measurements of the release kinetics are time consuming, require large drug loads and depend on many parameters including the geometry of the dosage form, the swelling capacity, and the type and amount of release medium [52,53]. Therefore, the use of alternative techniques that provide a reliable determination of the diffusivity coefficient of the drug into the hydrogel overcoming the previous issues is highly recommended. Diffusion-ordered correlation spectroscopy (DOSY) methods used under magic angle spinning (MAS) of the gel sample is a novel approach suitable for this purpose. Indeed, high-resolution (HR) spectra are obtained by orienting the sample at the so-called magic angle (54.78° with respect to the z direction), and spinning the sample at a moderate rate (typically 4 KHz). Magnetic field gradients are also applied along the magic angle axis. This experimental set-up averages the anisotropic interactions (dipole–dipole interactions and chemical shift anisotropy), leading to well-resolved spectra even for small molecules entrapped in a gel matrix. This methodology opens the possibility to study the diffusion motion of drugs loaded onto heterogeneous gel-like systems and to evaluate in depth the motion regime of the drug in the polymeric gel, including the detection of non-fickian diffusion regimes and related anomalous diffusion behaviours [44,54].

In this work the diffusion process of Ibuprofen (IB), chosen as model molecule, into the TOUS-CNFs/Ca^2+^ hydrogel matrix was investigated. IB is, indeed, a well-known and widely used anti-inflammatory drug. It can be administered to the patients by different routes including enteral, parenteral and topical administration [55,56]. The transdermal administration of IB with hydrogels is routinely employed for relieving pains and inflammations associated with backache, rheumatic pain, and muscular aches. New formulations are continuously developed to improve skin permeability, bioavailability, and safety [56]. Recently, hydrogels of nanocellulose loaded with IB have been the subject of a comparative study with marketed products [57].

**Figure 5:**
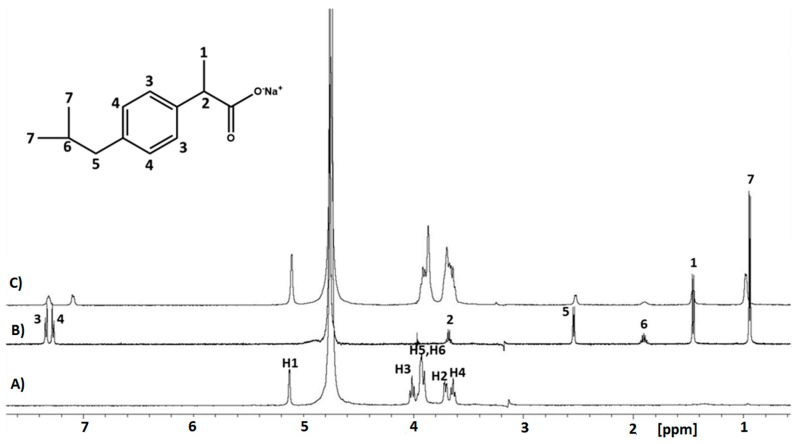
^1^H high-resolution magic angle spinning (HR-MAS) NMR spectra of: **A**) β-cyclodextrin (β-CD); **B**) ibuprofen (IB); **C**) IB /β-CD (1:1) complex loaded in **C1** hydrogel (details in Supplementary Materials Figures S2–S4).

IB was incorporated into the **C1** hydrogel either as a pure molecule or as a 1:1 complex with β-CD to investigate the possible effect of the host cavitand on the diffusion coefficient of IB. The use of CDs is, indeed, very common in pharmaceutical technology for the solubilisation and stabilization of several drugs [58]. The ^1^H HR-MAS spectra of β-CD, IB and IB/β-CD (1:1) complex loaded in the **C1** hydrogel are reported in Figure 5 together with line assignment. The molecular formula of ibuprofen and the atom numbering are also shown. The presence of the IB/β-CD (1:1) complex into the hydrogel is well visible (Figure 5c). In particular, NMR signals assignable to the methyl groups H7 and the aromatic H4 undergo the most noticeable chemical shift variation, with a frequency shift of 53 and 55 Hz, respectively. The expanded aliphatic and aromatic regions of the spectrum are reported in Figures S1–S2 for clarity. Moreover, complexation induced chemical shift variation is also observed for the β-CD resonances H3 and H5, consistent with the inclusion of the aromatic moiety of IB drug in the lipophilic cavity of cyclodextrin (Figure S3).

**Figure 6:**
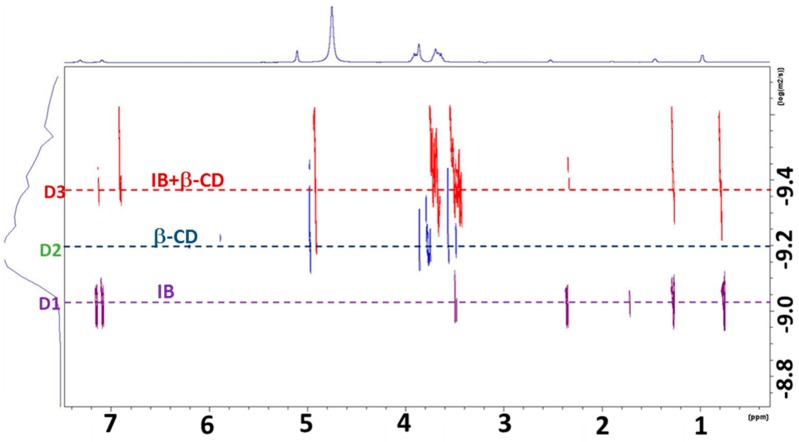
Overlap of diffusion-ordered correlation spectroscopy (DOSY) maps of: IB loaded in **C1** hydrogel (purple), β-CD loaded in **C1** hydrogel (blue) and IB/β-CD (1:1) complex loaded in **C1** hydrogel (red).

The presence of the inclusion complex is further highlighted by the diffusion-ordered spectroscopy (DOSY) NMR experiments. The DOSY experiment is based on the gradient echo approach. The NMR pulse sequence is designed in such a way that a spatial encoding of the diffusing molecules can be achieved, leading to a detectable magnetization attenuated due to partial refocusing. In turn, the signal attenuation can be related to the diffusion coefficient of the different diffusing molecular species. The resulting DOSY maps can be interpreted as a pseudo-2D NMR experiment, with chemical shifts and diffusion coefficients plotted, respectively, along x and y axis. [52,59]. The DOSY map of our systems, reported in Figure 6, is the superposition of three different DOSY experiments carried out on the IB-loaded hydrogel (purple trace), β-CD-loaded hydrogel (blue trace) and IB/β-CD (1:1) complex loaded hydrogel (red trace). The latter sample was investigated with the final aim to verify the possibility to modulate the IB diffusion rate in the hydrogel by increasing the hindrance of the guest.

The dotted horizontal lines are a guide for eye showing the three different diffusion coefficients, D1 (9.0 × 10^−10^ m^2^ s^−1^), D2 (6.3 × 10^−10^ m^2^ s^−1^) and D3 (4.3 × 10^−10^ m^2^ s^−1^), characterizing the free diffusion in the gel of Ibuprofen, β-CD and their inclusion complex, in the order. In this latter system, the signals of β-CD and IB show the same diffusion coefficient indicating an increased hydrodynamic radius of the diffusing species, in turn consistent with the formation of a supramolecular complex, with the two components in fast exchange on the NMR timescale. Furthermore, the D3 value of the complex is in turn smaller than the diffusion coefficient of two components (D1 and D2) loaded in the same gel medium (purple and blue traces). Therefore, IB is entrapped in the β-CD cavity even in **C1** hydrogel. Due to the lower diffusion coefficient of IB/β-CD (1:1) complex in **C1** hydrogel with respect to the pure IB molecule, the drug release process from the hydrogel could be modulated in a different way. In fact, in place of acting on the hydrogel characteristics (the optimization of the rheological properties by a modification of the hydrogel composition does not necessarily warrant an optimal drug diffusion process) it is possible to play on the non-covalent interactions between the carrier (β-CD in our case) with the hydrogel matrix. It is worth mentioning the fact that the formation of the IB/β-CD occurs in a reversible way and is driven by non-covalent interactions. As a consequence, the modulation of the transport properties of the active compound occurs without the formation or breaking of chemical bonds, thus preserving the biological activity of the drug. In a further perspective, the carrier host could be specifically functionalized in order to slow-down the diffusion process by introducing suitable groups that provide stronger interactions with the hydrogel. In this regard, a large number of modified CDs are already commercially available and capable to provide host-guest complexes with several drugs [58]. Moreover, new methods for the selective chemical functionalization of CDs have been developed and reported in literature [60].

### 3.3. Cytocompatibility of 2,2,6,6-Tetramethylpiperidine 1-oxyl (TEMPO)-Oxidized and Ultra-Sonicated Cellulose Nanofibers (TOUS-CNFs)/Ca^2+^ Hydrogels

In order to use **C1** as injectable drug-delivery system, a set of specific tests were carried out including the evaluation of its stability in the culture medium and its cytocompatibility.

**Figure 7:**
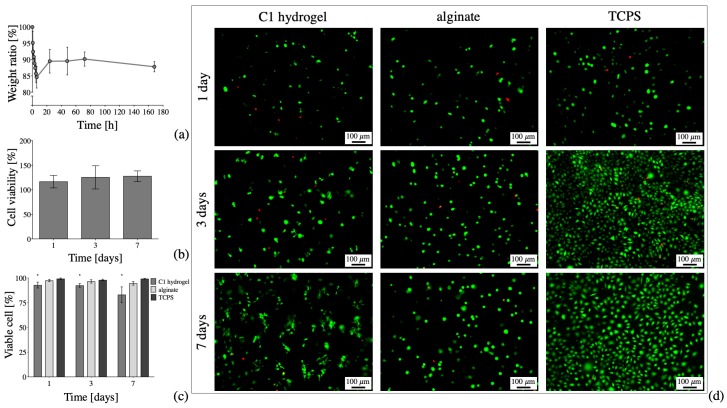
In vitro tests of **C1** hydrogels. (**a**) Percentage weight ratio of **C1** hydrogel immersed in culture medium at 37 °C, up to 7 days (i.e., 168 h). (**b**) Percentage L929 cell viability of cell cultured in culture medium eluates in contact with **C1** for 1, 3 and 7 days, compared to cells cultured in culture medium (i.e., control). (**c**,**d**) In vitro direct cytocompatibility tests of cells embedded in **C1**, in alginate/Ca^2+^ hydrogel (i.e., hydrogel control) and seeded on tissue culture plastic (i.e., tissue culture plastic (TCPS) as control): (**c**) percentage of viable L929 cells (* *p* < 0.05, comparing a sample to the others at the same time point) and (**d**) representative LIVE/DEAD staining images (scale bar: 100 µm).

With regard to the hydrogel stability, the percentage weight ratio (Equation (1)) of the **C1** hydrogel immersed in culture medium at 37 °C for 7 days is shown in Figure 7a. After a decrease in weight (i.e., 15% weight loss) up to 6 h of incubation, the C1 hydrogel reaches a stable weight (i.e., weight ratio = 90% vs. initial weight) up to 7 days of immersion in culture medium. The initial decrease in weight might be ascribed to a further crosslink induced by the presence of polyvalent ions (Ca^2+^ and Fe^3+^) in the DMEM medium, with a consequent slight shrink of the hydrogel. Indeed, it is reported in literature that Fe^3+^ can induce the formation of stable TOUS-CNFs hydrogels with higher storage module respect to Ca^2+^ [34]. After that first initial weight loss, the weight ratio of **C1** evidences the stability of the crosslinked structure, thus proving the efficiency of the Ca^2+^-driven crosslinking in obtaining a stable TOUS-CNFs hydrogel.

In order to assess the cytocompatibility of **C1,** indirect and direct cytotoxicity were evaluated. For the first case, the percentage cell viability of L929 cell cultured in presence of culture medium eluates extracted in contact with **C1** for 1, 3 and 7 days is shown in Figure 7b. For all the time points of the indirect in vitro cytotoxicity test considered, the measured cell viability is in any case higher than 70%, thus proving the absence of cytotoxic effects, in accordance with the standard ISO 10993-5 [51]. No differences in percentage cell viability were detected considering the three time points (*p* > 0.05). These in vitro results are in accordance with the low mortality rate described by other authors [61] for embryonic zebrafishes exposed to TOUS-CNFs, thus supporting the safety in using TOUS-CNFs in contact with cells. In particular, in our study, percentage cell viability measured for cells cultured with **C1** eluates was slightly higher than cells cultured in culture medium without TOUS-CNFs samples, for all the considered time points. This result is in accordance with the percentage cell viability described by other authors [62] that cultured lung epithelial alveolar cells (A549) in the presence of low concentrations of TOUS-CNFs. In particular, the authors demonstrate that cells directly exposed to low concentrations of TOUS-CNFs are characterized by increased proliferation, thus leading to a percentage of cell viability higher than the control (i.e., cells cultured in culture medium without TOUS-CNFs).

**Figure 8:**
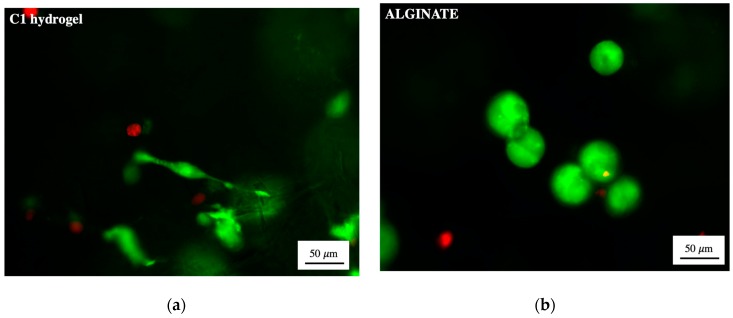
LIVE/DEAD staining of cells embedded in 2% *w*/*v* after 7 days of incubation (**a**) **C1** hydrogel and (**b**) alginate/Ca^2+^ hydrogel (scale bar: 50 µm).

Once verified the absence of indirect cytotoxic effects, cells were directly embedded in **C1** hydrogel and their viability checked. The percentage number of viable cells after 1, 3 and 7 days of culture is shown in Figure 7c, in comparison to the percentage of viable cells embedded in alginate/Ca^2+^ hydrogels and seeded on the bi-dimensional TCPS, as control. After 1, 3 and 7 days of culture in **C1** hydrogel, the percentage number of viable cells is higher than 70%, as qualitatively confirmed by the presence of green cells in the representative LIVE/DEAD staining images in Figure 7d. Similarly, the percentage cell viability of cells embedded in alginate (i.e., used as control for the similar Ca^2+^-driven crosslinking mechanism) and cultured on TCPS was higher than 70% for all the three time points considered. The high percentage of viable cells in **C1** proves the possibility of embedding viable cells in TOUS-CNFs/Ca^2+^ hydrogels, even though the percentage number of viable cells embedded in **C1** was lower (*p* < 0.05) compared to those embedded in alginate and those seeded on TCPS. The percentage of viable cells in **C1** at the different considered time points (i.e., 1 vs. 3 vs. 7 days), did not evidence a statistical difference (*p* > 0.05), thus proving the possibility of culturing viable cells, as demonstrated by other authors who used TOUS-CNFs microbeads to release viable encapsulated cells [63,64]. Moreover, after 1 and 3 days of culture, L929 cells were qualitatively characterized by a spherical morphology when embedded and cultured either in **C1** or in alginate/Ca^2+^ hydrogels [65]. After 7 days of culture, we evidenced a qualitative difference in the morphology of cells cultured in **C1**, which appeared more elongated, compared to cells embedded inside crosslinked alginate, still characterized by a more spherical morphology, as confirmed by higher magnification images (Figure 8). This difference can be given by the 3D structure of TOUS-CNFs, which mimic the extracellular matrix (ECM) structure and subsequently promote cell spreading, as demonstrated for spread cells cultured on CNF films [66]. Thus, despite the debate on the safety of using nanocellulose fibers for biomedical applications [67], our data suggest the absence of indirect cytotoxic effects together with the possibility of embedding viable cells inside TOUS-CNFs/Ca^2+^ hydrogels. These results represent a promising starting point for the use of TOUS-CNFs-based hydrogels for the production of systems with embedded cells (e.g., microbeads, bioprinted structures).

## 4. Conclusions

This work proves the suitability of TOUS-CNFs hydrogels for a broad potential range of applications in the biomedical field. As demonstrated by the rheology measurements, the physico-chemical properties of the material can be easily fine-tuned by the addition of a proper amount of a polyvalent cation (i.e., Ca^2+^) leading to the formation of self-sustaining hydrogels. Such materials could find suitable application for the controlled release of drugs. In this regard, the diffusion coefficient of ibuprofen into an optimized gel matrix (**C1**), both as free molecule and as 1:1 complex with β-CD, was measured by DOSY HR-MAS NMR experiments. The inclusion of the drug into the β-CD cavity led to a reduction of the diffusivity (4.3 × 10^−10^ m^2^ s^−1^) compared to the free molecule (9.0 × 10^−10^ m^2^ s^−1^). This result offers a different way to modulate the drug diffusion by playing with the non-covalent interactions between a carrier (β-CD in our case) and the hydrogel matrix without further modification of the hydrogel properties. Indeed, the physico-chemical properties of the carrier can be modified independently by the introduction of suitable functional groups. Finally, the absence of indirect cytotoxic effects is demonstrated by exposing L929 murine fibroblast cells towards eluates extracted in contact with **C1** for 1, 3 and 7 days. The measured cell viability is higher than 70% proving the absence of cytotoxic effects. The absence of cytotoxicity was assessed by directly embedding the L929 murine fibroblast cells in **C1** hydrogel and checking their viability. After 1, 3 and 7 days of culture, the percentage number of viable cells is higher than 70%. Similarly, the percentage cell viability of cells embedded in alginate and cultured on TCPS was higher than 70% for all the three time points considered.

## Figures and Tables

**Table 1:** Samples of TOUS-CNFs stock dispersions.

ID	TOUS-CNFs Concentration (*w*/*v*) (%)	Cellulose (g)	NaOH 0.1 M (mL)	Water (mL)
A	0.5	0.2	3.02	36.98
B	1	0.4	6.04	33.96
C	2	0.8	12.08	27.92
D	4	1.6	24.16	15.84

**Table 2:** Samples of TOUS-CNFs/Ca^2+^ stock dispersions.

ID	Cellulose Concentration (w/v) (%)	CaCl_2_ Final Concentration (mM)	TOUS-CNFs Dispersion (mg)	CaCl_2_ Solution 100 mM (µL)	Water (µL)
A1	0.5	10	450	50	0
A2	0.5	5	450	25	25
A3	0.5	2	450	10	40
A4	0.5	0	450	0	50
B1	1	10	450	50	0
B2	1	5	450	25	25
B3	1	2	450	10	40
B4	1	0	450	0	50
C1	2	10	450	50	0
C2	2	5	450	25	25
C3	2	2	450	10	40
C4	2	0	450	0	50

## Supplementary Materials

Supplementary materials are available online at https://www.mdpi.com/1996-1944/13/1/183/s1. Figure S1: Ibuprofen chemical structure, Figure S2: Zoom of ^1^H high-resolution magic angle spinning nuclear magnetic resonance (HR-MAS) spectra of: (**a**) ibuprofen (IB); (**b**) IB/β-cyclodextrin (β-CD) (1:1) complex loaded in **C1** hydrogel, Figure S3: Zoom of ^1^H HR-MAS spectra of: (**a**) IB; (**b**) IB/β-CD (1:1) complex loaded in **C1** hydrogel, Figure S4: Zoom of ^1^H HR-MAS spectra of: (**a**) β-CD; (**b**) IB/β-CD (1:1) complex loaded in **C1** hydrogel.
