# Supplementary material for: TEMPO-Nanocellulose/Ca2+ Hydrogels: Ibuprofen Drug Diffusion and In Vitro Cytocompatibility
Source: Materials (Basel). 2020 Jan 2;13(1):183. doi: 10.3390/ma13010183 (PMC6981511; doi:10.3390/ma13010183)
Supplement: Supplementary file 1 [file materials-13-00183-s001.pdf]

materials
MDPI

Supplementary Materials

Ibuprofen Diffusion and L929 Cells' Encapsulation in TEMPO-Nanocellulose Hydrogels

Andrea Fiorati [1,2,*], Nicola Contessi Negrini [1,2], Elena Baschenis [1], Lina Altomare [1,2], Silvia Faré [1,2], Alberto Giacometti Schieroni [3], Daniele Piovani [3], Raniero Mendichi [3], Monica Ferro [1], Franca Castiglione [1], Andrea Mele [1,3], Carlo Punta [1,2] and Lucio Melone [1,2,*]

[1] Department of Chemistry, Materials, and Chemical Engineering "G. Natta"—Politecnico di Milano, Piazza Leonardo da Vinci 32, Milano I-20133, Italy; nicola.contessi@polimi.it (N.C.N.); baschenis.e@gmail.com (E.B.); lina.altomare@polimi.it (L.A.); silvia.fare@polimi.it (S.F.); monica.ferro@polimi.it (M.F.); franca.castiglione@polimi.it (F.C.); andrea.mele@polimi.it (A.M.); carlo.punta@polimi.it (C.P.)
[2] INSTM, National Consortium of Materials Science and Technology, Local Unit Politecnico di Milano, Milano 20133, Italy
[3] Istituto di Scienze e Tecnologie Chimiche (SCITEC-CNR), Via A. Corti 12, Milano 20133, Italy; giacometti@ismac.cnr.it (A.G.S.); piovani@ismac.cnr.it (D.P.); rmendichi@libero.it (R.M.)
* Correspondence: andrea.fiorati@polimi.it (A.F.); lucio.melone@polimi.it (L.M.)

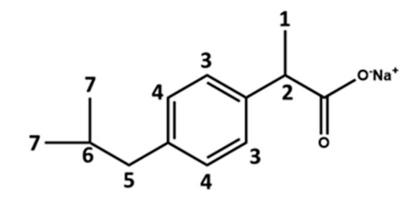

Figure S1. Ibuprofen chemical structure.

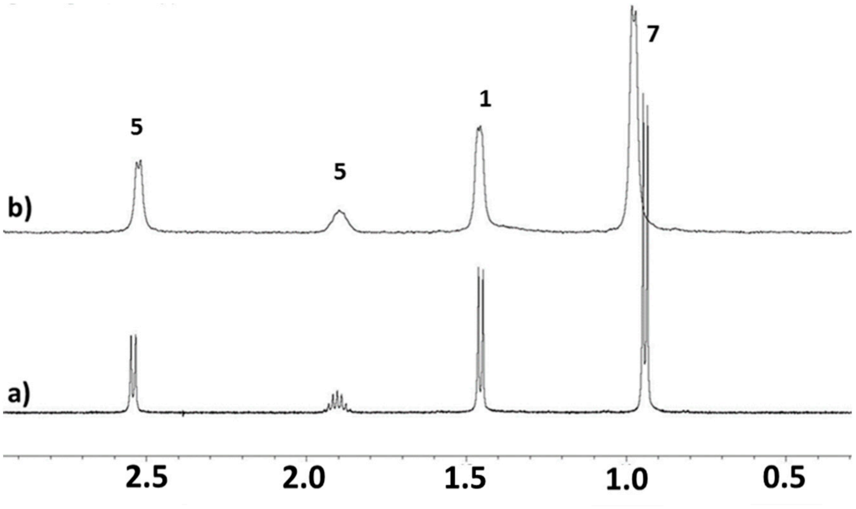

Figure S2. Zoom of ^{1}H high-resolution magic angle spinning nuclear magnetic resonance (HR-MAS) spectra of: (**a**) ibuprofen (IB); (**b**) IB/β-cyclodextrin (β-CD) (1:1) complex loaded in **C1** hydrogel.

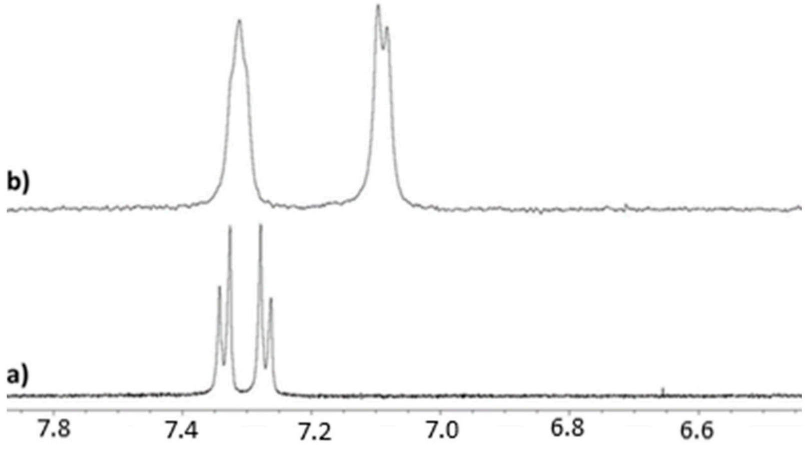

Figure S3. Zoom of ^{1}H HR-MAS spectra of: (**a**) IB; (**b**) IB/β-CD (1:1) complex loaded in **C1** hydrogel.

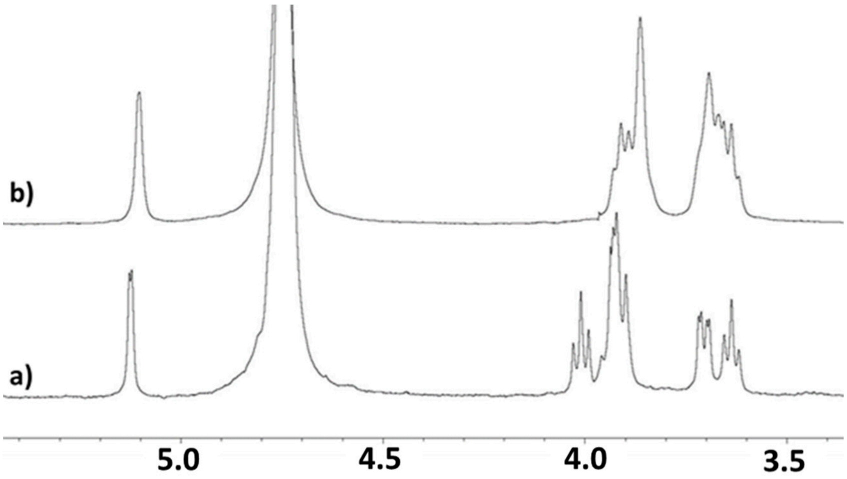

Figure S4. Zoom of ^{1}H HR-MAS spectra of: (**a**) β-CD; (**b**) IB/β-CD (1:1) complex loaded in **C1** hydrogel.

© 2020 by the authors. Submitted for possible open access publication under the terms and conditions of the Creative Commons Attribution (CC BY) license (http://creativecommons.org/licenses/by/4.0/).
